# CHCHD2 links mitochondrial dysfunction and α-synuclein misfolding in Parkinson’s disease

**DOI:** 10.1016/j.tins.2026.02.002

**Published:** 2026-03

**Authors:** Derek Narendra, Brent J. Ryan

**Affiliations:** 1 Mitochondrial Biology and Neurodegeneration Unit, Neurogenetics Branch, National Institute of Neurological Disorders and Stroke, National Institutes of Health, Bethesda, MD 20892, USA; 2 Oxford Parkinson’s Disease Centre, Department of Physiology, Anatomy and Genetics, University of Oxford, Oxford, UK; 3 Kavli Institute for Nanoscience Discovery, University of Oxford, Oxford, UK

**Keywords:** dopaminergic neurons, mitochondrial metabolism, disease stratification, protein aggregation, oxidative stress, neurodegeneration

## Abstract

Parkinson’s disease comprises multiple biological subtypes and a heterogeneous clinical course. A recent study by Liao *et al*. identifies CHCHD2 mutations as a mitochondrial entry point that links metabolic dysfunction to α-synuclein pathology. These findings highlight how rare sporadiclike monogenic forms of Parkinson’s disease may inform mechanistic and therapeutic stratification.

## Main text

Parkinson’s disease is increasingly understood as a syndrome with multiple biological entry points that converge on differing clinical courses, ranging from a limited movement disorder to diffuse neurodegeneration with cognitive impairment. Given ‘idiopathic’ Parkinson’s disease may be a composite of genetic, molecular, and clinical subtypes, this heterogeneity raises a practical question for patient stratification: which monogenic forms of Parkinson’s disease capture mechanisms shared across major sporadic subgroups, and which instead define clinically overlapping, but biologically distinct, forms of Parkinsonism? *In vivo* models that recapitulate key Parkinson’s disease phenotypes, including α-synuclein aggregation, mitochondrial dysfunction, and loss of dopaminergic neurons of the midbrain, are important for understanding disease mechanisms and testing potential therapeutics.

In a recent study, Liao *et al.* [1] addressed this need by generating a knock-in mouse model for a monogenic form of Parkinson’s disease caused by a dominant mutation (T61I) in the mitochondrial protein CHCHD2. Although rare, the phenotype of CHCHD2 patients, including the presence of midbrain and cortical Lewy bodies, is similar to idiopathic Parkinson’s disease [2]. Coiled-coil-helix-coiled-coil-helix domain-containing protein 2 (CHCHD2) is a small, mitochondria-localized protein enriched in energy-demanding neurons, where it supports respiratory function and mitochondrial ultrastructure, providing a biologically plausible link between mitochondrial dysfunction and idiopathic-like disease. Although knockout of CHCHD2 and its paralog CHCHD10 is well tolerated in mice, dominant mutations in either protein are linked to protein misfolding and cellular death via a toxic gain-of-function mechanism 3., 4, 5. In a neuropathologic report of an affected CHCHD2 T61I carrier, CHCHD2 aggregates were observed in affected brain regions, hinting at a similar toxic gain-of-function mechanism [2].

Consistent with a gain-of-function mechanism, Liao *et al.* found that the T61I mutation does not alter total CHCHD2 abundance but instead drives an age-dependent redistribution into punctate, insoluble structures, suggestive of intramitochondrial aggregation [1]. These puncta contain CHCHD10 and are associated with ultrastructural changes, including increased mitochondrial swelling in substantia nigra dopaminergic neurons. As in neuropathological material, CHCHD2 aggregation is associated with α-synuclein misfolding, directly linking the CHCHD2 axis to synuclein biology. In CHCHD2 T61I mice, total α-synuclein and phosphorylated α-synuclein (pS129) are increased in the substantia nigra, while age-dependent cortical aggregation suggests that mutant CHCHD2 accelerates synuclein pathology beyond the nigrostriatal system. Spatial transcriptomics further showed that CHCHD2 and CHCHD10 transcripts, like SNCA, are enriched in vulnerable ventral dopaminergic neurons, consistent with higher mitochondrial demand. Early human Parkinson’s disease tissue similarly shows selective reduction of CHCHD2 in ventral dopaminergic neurons, suggesting preferential loss of low-expressing neurons or active repression during stress.

The authors went on to show that mitochondrial damage, potentially driven by CHCHD2 aggregation, disrupts energy metabolism in the brain. U-^13^C glucose labeling revealed increased flux through glycolytic and pentose phosphate pathways in CHCHD2 T61I knock-in mice, with relatively little incorporation into TCA cycle metabolites, consistent with impaired oxidative phosphorylation. This shift toward glycolytic metabolism was also evident systemically by comprehensive laboratory animal monitoring system (CLAMS) analysis. These metabolic changes were accompanied by altered abundance of complex I- and III-associated proteins and reorganization of respiratory chain networks, as determined by complexomics. Despite preserved complex I enzymatic activity, increased reactive oxygen species production and mitochondrial morphological abnormalities suggest inefficient oxidative phosphorylation driving metabolic compensation. Notably, these metabolic signatures overlapped with Parkinson’s disease dementia and incidental Lewy body disease, reinforcing evidence for metabolic dysfunction in Parkinson’s disease. Convergent findings from monogenic disorders affecting glycolysis and detoxification of reactive intermediates (PARK7 and PGK1), as well as mitochondrial quality control and function (PRKN, PINK1, WARS2, TWNK, and POLG), suggest that enhanced glycolysis may represent a compensatory and potentially druggable pathway [6].

In CHCHD2 T61I mice, metabolic disruption does not cause overt dopaminergic neuron loss but produces mild behavioral and neurophysiological changes, including impaired D2 autoreceptor-mediated autoinhibition, hyperexcitability, and age-dependent dopamine reduction, indicating early functional vulnerability preceding cell loss [1]. The absence of a more robust phenotype is not unexpected, as multiple genetic Parkinson’s disease mouse models, including those targeting PRKN, PINK1, DJ1, and VPS13C, show substantial neuronal compensation despite causing early-onset Parkinson’s disease in humans. Together, the findings by Liao *et al*. demonstrate that intramitochondrial CHCHD2 misfolding is linked to widespread metabolic changes, α-synuclein pathology, and subtle dopamine neuron dysfunction.

Importantly, these findings are consistent with CHCHD2 misfolding driving Parkinson’s disease pathogenesis in a manner analogous to dominant mutations in its paralog CHCHD10, which cause neuromuscular disorders ranging from frontotemporal dementia (FTD) and amyotrophic lateral sclerosis (ALS) to mitochondrial myopathy 3., 4, 5. In the CHCHD2 T61I model, mutant CHCHD2 drives coaccumulation and coaggregation of CHCHD10 into punctate structures, supporting secondary recruitment of CHCHD10 [7]. Conversely, certain CHCHD10 variants, such as S59L, induce coaggregation with CHCHD2, shifting both proteins into insoluble fractions [4]. Why mutations in these closely related proteins give rise to distinct neurodegenerative diseases with different regional vulnerabilities—Parkinson’s disease versus FTD–ALS—remains unresolved. Notably, aggregation of CHCHD2 in Parkinson’s disease and CHCHD10 in FTD–ALS appears selective to affected neuronal populations 2, 8, suggesting mutation-specific, cell-type-dependent toxic misfolding.

A notable strength of the study by Liao *et al*. is its deeply integrated phenotyping across physiology, ultrastructure, metabolism, spatial transcriptomics, and mitochondrial protein networks, meeting a high bar for *in vivo* Parkinson’s disease models and enabling more precise mechanistic stratification. Building on this depth, the authors provide a detailed examination of a mitochondrial genetic entry point into Parkinson’s disease and a framework for relating such mechanisms to the heterogeneous clinical and pathological features of sporadic Parkinson’s disease. The CHCHD2 p.T61I model supports a nuanced view in which mitochondrial dysfunction is not uniform, defining a pathway that does not phenocopy the nigropathy seen in PINK1 or PRKN deficiency, yet converges on α-synuclein pathology and dopaminergic dysfunction reminiscent of sporadic disease. This contrasts with α-synuclein overexpression models, where mitochondrial impairment and oxidative stress coexist with complex I deficits and TWNK or POLG mutations, which cause severe complex I deficiency in human dopamine neurons without Lewy body formation 9, 10. Together, these distinctions argue that mitochondrial dysfunction in Parkinson’s reflects overlapping but divergent pathways that intersect with α-synuclein biology and that applying similarly deep phenotyping across genetic and sporadic models will be essential to define how distinct entry points shape the heterogeneous clinical landscape of Parkinson’s disease.

## References

[1] Liao S.-C. (2025). CHCHD2 mutant mice link mitochondrial deficits to PD pathophysiology. Sci. Adv..

[2] Ikeda A. (2019). Mutations in CHCHD2 cause α-synuclein aggregation. Hum. Mol. Genet..

[3] Liu Y.-T. (2020). Loss of CHCHD2 and CHCHD10 activates OMA1 peptidase to disrupt mitochondrial cristae phenocopying patient mutations. Hum. Mol. Genet..

[4] Anderson C.J. (2019). ALS/FTD mutant CHCHD10 mice reveal a tissue-specific toxic gain-of-function and mitochondrial stress response. Acta Neuropathol..

[5] Genin E.C. (2019). Mitochondrial defect in muscle precedes neuromuscular junction degeneration and motor neuron death in CHCHD10(S59L/+) mouse. Acta Neuropathol..

[6] Cai R. (2019). Enhancing glycolysis attenuates Parkinson’s disease progression in models and clinical databases. J. Clin. Invest..

[7] Huang X. (2018). CHCHD2 accumulates in distressed mitochondria and facilitates oligomerization of CHCHD10. Hum. Mol. Genet..

[8] Keith J.L. (2020). Neuropathologic description of CHCHD10 mutated amyotrophic lateral sclerosis. Neurol. Genet..

[9] Ryan B.J. (2021). REST protects dopaminergic neurons from mitochondrial and alpha-synuclein oligomer pathology in an alpha synuclein overexpressing BAC-transgenic mouse model. J. Neurosci..

[10] Palin E.J.H. (2013). Mesencephalic complex I deficiency does not correlate with parkinsonism in mitochondrial DNA maintenance disorders. Brain.

